# Importance of Cardiovascular Magnetic Resonance Applied to Congenital Heart Diseases in Pediatric Age: A Narrative Review

**DOI:** 10.3390/children11070878

**Published:** 2024-07-19

**Authors:** Sara Moscatelli, Alice Pozza, Isabella Leo, Jessica Ielapi, Alessandra Scatteia, Sofia Piana, Annachiara Cavaliere, Elena Reffo, Giovanni Di Salvo

**Affiliations:** 1 Centre for Inherited Cardiovascular Diseases, Great Ormond Street Hospital, London WC1N 3JH, UK; 2 Institute of Cardiovascular Sciences, University College London, London WC1E 6BT, UK; 3 Division of Paediatric Cardiology, Department of Women and Children’s Health, University Hospital of Padua, 35128 Padua, Italysofia.piana@studenti.unipd.it (S.P.); elena.reffo@aopd.veneto.it (E.R.); 4 Experimental and Clinical Medicine Department, University Magna Graecia of Catanzaro, 88100 Catanzaro, Italy; i.leo@unicz.it (I.L.); jessica.ielapi@studenti.unicz.it (J.I.); 5 Advanced Cardiovascular Imaging Unit, Clinica Villa dei Fiori, 80011 Acerra, Italy; alessandra.scatteia@villadeifioriacerra.com; 6 Department of Medical, Motor and Wellness Sciences, University of Naples ‘Parthenope’, 80134 Naples, Italy; 7 Pediatric Radiology, Neuroradiology Unit, University Hospital of Padua, 35128 Padua, Italy; annachiara.cavaliere@aopd.veneto.it

**Keywords:** congenital heart disease (CHD), magnetic resonance (CMR), cardiac magnetic technique

## Abstract

Congenital heart diseases (CHDs) represent a heterogeneous group of congenital defects, with high prevalence worldwide. Non-invasive imaging is essential to guide medical and surgical planning, to follow the patient over time in the evolution of the disease, and to reveal potential complications of the chosen treatment. The application of cardiac magnetic resonance imaging (CMRI) in this population allows for obtaining detailed information on the defects without the necessity of ionizing radiations. This review emphasizes the central role of CMR in the overall assessment of CHDs, considering also the limitations and challenges of this imaging technique. CMR, with the application of two-dimensional (2D) and tri-dimensional (3D) steady-state free precession (SSFP), permits the obtaining of very detailed and accurate images about the cardiac anatomy, global function, and volumes’ chambers, giving essential information in the intervention planning and optimal awareness of the postoperative anatomy. Nevertheless, CMR supplies tissue characterization, identifying the presence of fat, fibrosis, or oedema in the myocardial tissue. Using a contrast agent for angiography sequences or 2D/four-dimensional (4D) flows offers information about the vascular, valvular blood flow, and, in general, the cardiovascular system hemodynamics. Furthermore, 3D SSFP CMR acquisitions allow the identification of coronary artery abnormalities as an alternative to invasive angiography and cardiovascular computed tomography (CCT). However, CMR requires expertise in CHDs, and it can be contraindicated in patients with non-conditional devices. Furthermore, its relatively longer acquisition time and the necessity of breath-holding may limit its use, particularly in children under eight years old, sometimes requiring anesthesia. The purpose of this review is to elucidate the application of CMR during the pediatric age.

## 1. Introduction

Congenital heart diseases (CHDs) represent the most prevalent group of congenital defects worldwide, exhibiting a prevalence of approximately 0.9% of liveborn children [1,2]. CHDs consist of abnormalities in the development of the heart and great vessels. They are divided in two main categories: cyanotic CHD (CCHD); and acyanotic CHDs. CCHD represents a cardiac emergency in the neonatal period because it is characterized by a right-to-left shunt, which allows deoxygenated blood to mix with the oxygenated blood of the vascular circuit. The acyanotic CHDs, on the other hand, can manifest as either obstruction or shunt lesions. Obstructive lesions may occur in the ventricular inflow tracts, outflow tracts, and in the great vessels, leading to proximal chamber hypertrophy and distal dilatation to the obstruction. Shunt lesions create abnormal communications between the left and right heart chambers, and include conditions such as atrial septal defect (ASD), ventricular septal defect (VSD), patent ductus arteriosus (PDA), and atrio-ventricular canal defects [2].

The development of prenatal screening, pediatric diagnostic techniques, and therapeutical innovations have contributed to increased survival among this heterogeneous group of patients, most of whom arrive in adulthood [3,4,5]. Advanced non-invasive imaging, providing anatomical and functional information, guides medical and surgical planning, and permits following the evolution of the disease over time, also revealing potential issues related to the chosen treatment [6,7]. Echocardiography is the most commonly used diagnostic technique for evaluating patients with CHD, with both pediatric and adult populations. However, it has significant limitations, particularly in patients with poor acoustic windows, and its imaging quality and interpretation are highly dependent on the skill and experience of the operator. Nowadays, cardiac magnetic resonance (CMR) is largely used, and overcomes echocardiographic limitation (Table 1), offering detailed information about the cardiac anatomy, function, flow, and tissue properties characteristics, as well as the evaluation of myocardial viability and perfusion without ionizing radiations. Nonetheless, CMR is currently widely available, although it requires high expertise in the CHD context, necessitating that the examination of these patients be performed in highly specialized and dedicated centers [8,9,10,11,12,13].

A general standard CMR protocol for evaluating CHDs in pediatric patients includes: real-time localization imaging in three planes without ECG gating, useful for anatomy and extracardiac structures; two-dimensional (2D) balanced steady-state free precession (bSSFP) cine sequence, to report the anatomy, size and function of the ventricles; the 2D phase contrast (PC) flow sequences, to permit the evaluation of vascular and valvular flow, although recently the four-dimensional flow CMR resonance (4DFlow CMR) imaging technique has allowed a comprehensive and detailed analysis of cardiovascular flow in a single free-breathing acquisition, providing both quantitative and qualitative data on flow patterns in the heart and great vessels; whole heart isotropic three-dimensional (3D) SSFP imaging, for vascular evaluation without contrast material administration and visualization of proximal and mid-coronary arteries; and MR angiography (MRA), for vascular evaluation [11,12,13,14,15,16,17,18,19]. Moreover, CMR permits tissue characterization by acquiring T1 and T2 mapping sequences, which uses the proton density of the tissue to identify areas of fibrosis, oedema, and fat [6,20,21]. In addition, late gadolinium enhancement (LGE) sequences identify myocardial inflammation and fibrosis, due to the accumulation and slower wash-out of gadolinium in the myocardial areas affected by these conditions. Early gadolinium enhancements (EGE) can also be acquired and provide information about thromboembolic formations [22,23].

The application of CMR in CHDs demands a high level of expertise, given the intricacies of CHDs’ anatomy and treatment. Moreover, CMR’s relatively longer acquisition time and requirement for breath-holding during scanning may pose challenges, particularly in pediatric patients under eight years old, sometimes necessitating general anesthesia to ensure successful imaging acquisition. Finally, this imaging technique can be contraindicated in patients with non-CMR conditional devices, even if in some centers these patients have been started to be scanned regardless [11,21].

The aim of this review is to clarify the role of CMR in the assessment of CHDs, highlighting its current practice and future perspective and revealing the possible challenges and limitations of this imaging technique.

## 2. Cardiovascular Magnetic Resonance Applications in the Congenital Heart Diseases Affecting the Pediatric Population

### 2.1. Cardiovascular Magnetic Resonance in Assessing Atrial Septal Defects, Ventricular Septal Defects, Patent Ductus Arteriosus, and Atrioventricular Septal Defects

Atrial septal defects (ASDs), ventricular septal defects (VSDs), and patent ductus arteriosus (PDA) are among the most common CHDs in adults. These anomalies can vary widely in presentation and impact cardiac function, making accurate and detailed imaging crucial for diagnosis and management. CMR offers distinct advantages over other imaging modalities, clarifying the diagnosis, establishing the defect’s location and size, demonstrating the need and the timing for intervention, and monitoring post-surgical corrections [24].

Different CMR techniques are useful for the characterization of patients with suspected cardiac shunts. First, thanks to the 2D bSFPP images, CMR can quantify left (L) and right ventricular (RV) volumes and functions, which can be challenging with 2D transthoracic echocardiography, especially for the RV, due to its complex anatomy [25]. In addition, CMR via 2D PC flow or 4D Flow images can assess forward stroke volume measurements at the main pulmonary artery (MPA) and proximal ascending aorta (Ao), estimating respectively the pulmonary flow (Qp) and the systemic flow (Qs) with the correspondent pulmonary-to-systemic circulation flow ratio (Qp/Qs). Information on cardiac volumes and functions and Qp/Qs ratio are fundamental for understating the hemodynamic significance of shunts guiding subsequent interventions [26].

#### 2.1.1. Atrial Septal Defects

ASDs represent communication between the atria. Transthoracic (TTE) and transesophageal echocardiography (TEE) remain the initial choice for evaluating ASDs to understand defect anatomy and guide percutaneous closure. However, it may not be sufficient in cases with complex anatomical abnormalities, especially for sinus venosus ASDs with an associated anomalous pulmonary venous return that needs an anatomical description of the pulmonary veins for repair procedure planning. CMR plays a vital role in defining the size, location, and hemodynamic impact of ASDs. Indeed, it can accurately measure the dimensions of the defects and assess the degree of right-sided volume overload, thanks to 2D SSFP sequences, and derive the Qp/Qs from the flow sequences as well as evaluating the presence and extent of associated complications, such as pulmonary hypertension. CMR should be strongly considered when: (1) the calculation of intracardiac shunting has been equivocal by echocardiography or interventional; (2) when RV dilation has been suspected on TTE without obvious detection of the anatomic defect; and (3) when associated anomalous pulmonary venous return is suspected [27,28]. In conclusion, CMR helps in setting an indication for ASDs closure when RV dilatation is detected or confirmed together with consensual increase in the Qp/Qs and the absence of pulmonary hypertension [6].

#### 2.1.2. Ventricular Septal Defects

**Figure 1:**
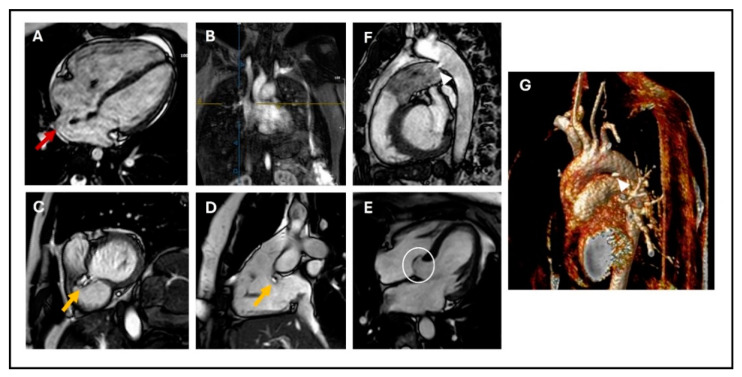
(**A**) 4-chamber cine SSFP image showing sinus venosus ASD (red arrow); (**B**) Angiographic reconstruction showing the right upper pulmonary artery draining into the superior vena cava (blue and yellow cross); (**C**) Basal short axis cine SSFP image; (**D**) sagittal RV three-chamber view showing perimembranous VSD (yellow arrow); (**E**) 4-chamber cine SSFP image showing aneurysmal formation of the basal septum involving adjacent septal leaflet of the tricuspid valve (white circle); (**F**) Sagittal cine SSFP image; and (**G**) MRA showing PDA: (arrow-heads). SSFP: steady-state free precession, ASD: atrial septal defect, RV: right ventricle, VSD: ventricular septal defect, PDA: patent ductus arteriosus.

VSDs are the most common CHDs at birth; they can be localized wherever in the septum (membranous, muscular, and outlet defects), but the most common are in the perimembranous area [29] (Figure 1C,D). VSDs tend to close spontaneously during childhood in 40% of the cases. They are defined as restrictive when they are small enough to create a pressure gradient between the ventricles, so that the pulmonary ventricle and pulmonary vasculature are protected from the systemic pressure. As for ASDs, echocardiography is the first-line imaging technique; however, while multiple 2D views of the septum can help evaluate the position of a defect, it can be challenging to visualize the real entirety of the VSD and accurately measure its dimensions [30]. CMR can overcome this limitation, providing precise measurements of defect size and location, thanks to 2D bSSFP and 3D reconstructions sequences, and it can give information about the hemodynamic consequences (LV dilatation, increased Qp/Qs with LV stroke volume greater than the RV stroke volume). Thanks to this information, CMR may be useful for determining the need for interventional closure or surgical repair, indicated by LV dilatation and increased Qp/Qs in the absence of pulmonary hypertension [24,26]. CMR can also visualize healed VSDs, which tend to be associated with the aneurysmal formation of the basal septum and sometimes involve adjacent septal leaflets of the tricuspid valve (Figure 1E) [6,20].

#### 2.1.3. Patent Ductus Arteriosus

Patent ductus arteriosus (PDA) is a fetal vascular structure connecting the proximal descending aorta to the roof of the main pulmonary artery [31]. Although essential in fetal life for the right ventricular ejection into the aorta, PDA typically closes spontaneously after birth. It is frequently observed in pre-term newborns and, depending on its persistence, size, and degree of left-to-right shunting, can cause significant pulmonary overload, leading to increased pulmonary vascular resistance and pulmonary hypertension. Indications for closure include symptomatic left-chamber dilation or dysfunction, with Eisenmenger’s syndrome posing a risk of increased morbidity and mortality.

Transcatheter closure is the established treatment of choice. Cardiac magnetic resonance imaging (CMR) provides a detailed visualization of PDA using techniques such as 2D balanced steady-state free precession (bSSFP) cine imaging, 3D SSFP reconstruction, or angiography sequences (Figure 1F,G). CMR also allows assessment of its hemodynamic consequences, including indirect methods for quantifying the shunt caused by PDA. These methods include calculating the difference between the left ventricular stroke volume and total systemic flow (superior vena cava + descending aorta), which should equal the ductal shunt volume, and using the Qp/Qs ratio, which typically shows less than 1 due to the left-to-right shunting effect [26,32].

#### 2.1.4. Atrio-Ventricular Septal Defects (AVSDs)

AVSDs are characterized by the absence of the muscular atrio-ventricular septum, inlet/outlet disproportion, abnormal lateral rotation of the postero-medial papillary muscle, and abnormal configuration of the atrioventricular valves. These defects can be complete or partial, often accompanied by varying degrees of atrio-ventricular valve abnormalities. Clinical presentation ranges from mild to severe depending on the size of the defect and associated cardiac anomalies.

Diagnosis typically relies on echocardiography, which assesses the anatomy and hemodynamics of the defect. Cardiac magnetic resonance imaging (CMR) complements echocardiography by providing detailed anatomical and functional information in diagnosing and characterizing atrioventricular septal defects (AVSDs). CMR enables precise assessment of the size, location, and extent of AVSDs, as well as the morphology and function of the atrioventricular valves [6,33]. It is also valuable in evaluating associated cardiac abnormalities such as anomalous pulmonary venous drainage and other complex structural anomalies commonly associated with AVSDs.

Furthermore, CMR facilitates accurate measurements of ventricular volumes and function, critical for surgical planning and assessing postoperative outcomes. Its most crucial role lies in post-surgical follow-up, as it is less commonly used before surgery. CMR plays a vital role in monitoring for complications such as residual shunts, atrio-ventricular valve dysfunction, and enlargement and dysfunction of the left and right ventricles, including left ventricular outflow tract obstruction [6,34].

### 2.2. Cardiovascular Magnetic Resonance in Assessing Conotruncal Congenital Heart Diseases

Conotruncal anomalies (CtA) are a group of CHDs that result from an altered pathway during embryogenesis, with abnormal formation and septation of the outflow tracts of the heart and the great vessels [35]. CtAs account for up to 25–30% of all non-syndromic CHDs and include tetralogy of Fallot (TOF), transposition of the great arteries (TGA), truncus arteriosus (TA), and double outlet right ventricle (DORV) [36]. When not appropriately diagnosed and managed, CtA might lead to significant morbidity and mortality [37]. Therefore, the need to find a proper diagnostic tool to adequately assess cardiac morphology, and at the same time to provide insight into ventricular performance [38].

#### 2.2.1. Dextro-Transposition of the Great Arteries (D-TGA)

Complete transposition of the great arteries (TGA), also referred to as dextro-transposition of the great arteries (D-TGA), is a developmental cardiac defect [39,40] characterized by atrio-ventricular concordance and ventriculo-arterial discordance [41,42]. D-TGA is defined “simple” in the case of no associated congenital anomalies, whereas it is categorized as “complex” in their presence [6]. CMR imaging is rarely performed in the preoperative setting [12,40,43]. Over the years, the surgical treatment for D-TGA has evolved from the atrial switch procedure (AtSO) to the arterial switch operation (ASO). Complex D-TGA is often repaired using the Rastelli procedure or its variants [6]. In post-surgical management, CMR is addressed to depict the most common complications and potential residual findings after these procedures and it is usually repeated every 2–4 years [12,38].

##### Atrial Switch Operation and the Role of CMR Imaging

**Figure 2:**
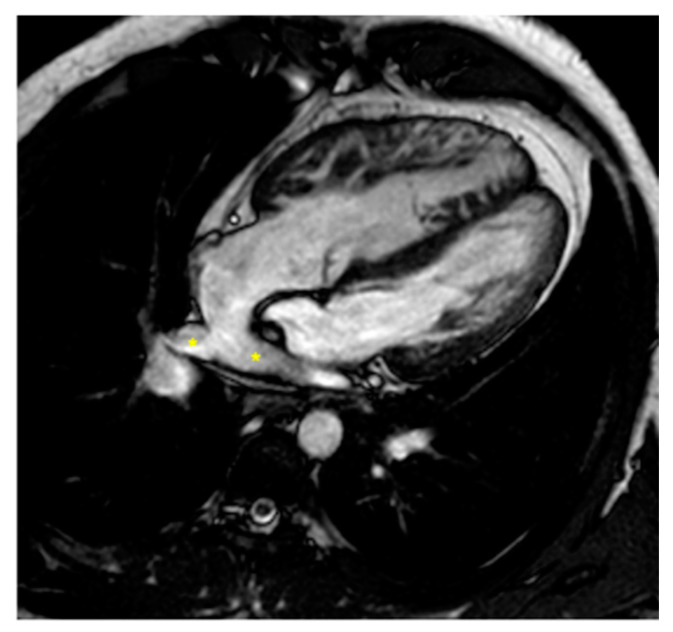
Cine bSSFP image of D-TGA post-atrial switch operation using the Senning technique. The image shows the pulmonary veins (*) being redirected through the baffle into the right atrium and then to the subaortic positioned sRV.

**Figure 3:**
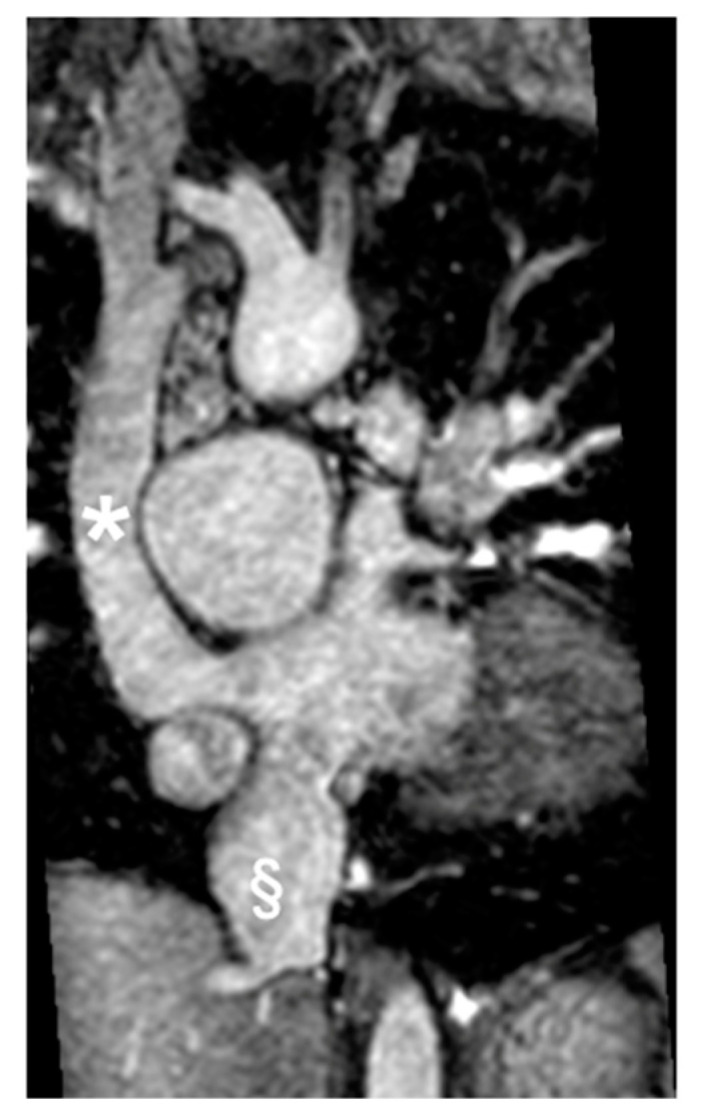
Cine bSSFP image of D-TGA following an atrial switch operation using the Senning technique. This image illustrates the pathway of the systemic veins, with the superior vena cava (*) and the inferior vena cava (§) shown.

Complications after AtSO include baffle stenosis or leaks, systemic tricuspid valve (TV) regurgitation, and systemic right ventricle (sRV) dysfunction, with potential pulmonary hypertension often identified during routine imaging [44,45,46]. CMR is the gold standard for assessing sRV issues, offering detailed insights into heart morphology, function, and ejection fraction [12,40,47,48,49,50,51] thanks to the cine sequences, and it is especially recommended for evaluating systemic TV and baffles (Figure 2 and Figure 3) that are well studied from the 2D bSSFP, 3D whole heart, and angiographies [12,40]. Tricuspid regurgitation (TR) often stems from annulus dilatation, valve prolapse, or medial cuspid tethering, with occasional surgical damage to the valve leaflets [52,53,54]. CMR is also essential for detecting and assessing the severity of leaks and stenosis in the interatrial baffle [12,40,45] obtained through flow sequences. Myocardial performance, particularly fibrosis detection, is crucial, as it correlates with adverse outcomes—up to 60% of sRV patients exhibit LGE [45,55,56].

##### Arterial Switch Operation and the Role of CMR Imaging

**Figure 4:**
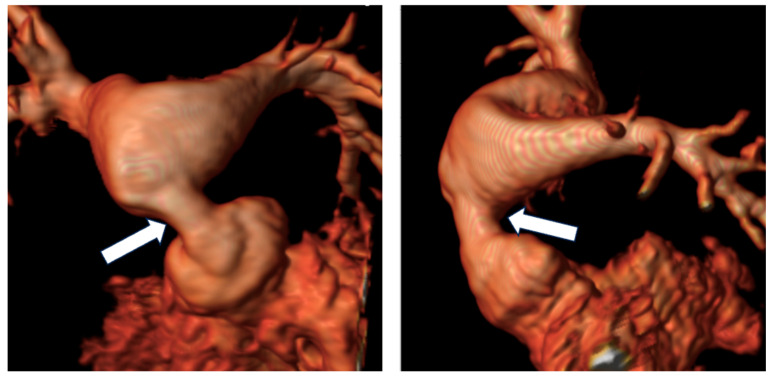
Reconstruction following angiographic sequences in TGA post-arterial switch operation. The image highlights a suprapulmonary stenosis (white arrows) at the level of the surgical suture of the switch with post-stenotic dilation.

Patients diagnosed with D-TGA post-1980s typically undergo the ASO, with late complications involving the great vessels, coronary arteries, and potential ventricular dilatation and dysfunction [40]. CMR imaging is crucial during long-term follow-up, particularly for assessing biventricular volumes, function, and morphology, as well as coronary artery and pulmonary artery stenosis (Figure 4) [6,12,57]. Despite normal ventricular volumes, decreased global longitudinal strain and LV torsion are noted [43,58]. CMR also evaluates myocardial perfusion, particularly in symptomatic patients, using vasodilator stress perfusion as a non-invasive test for ischemia and coronary obstruction [12]. It is essential for detecting myocardial scarring with LGE and should be repeated based on initial findings and symptoms [11,12,38,40,59,60].

##### Rastelli Procedure and the Role of CMR Imaging

**Figure 5:**
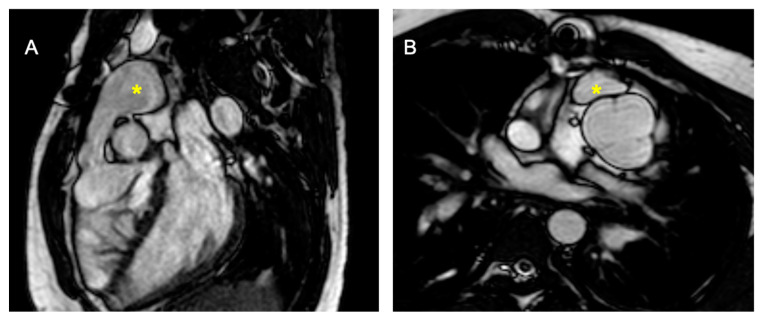
D-TGA with pulmonary stenosis/atresia post-placement of the right ventricle to pulmonary artery (RV-PA) conduit. (**A**) shows a sagittal section, and (**B**) displays a transverse section, both highlighting the pulmonary conduit (marked with *).

The Rastelli procedure and its variants are favored for D-TGA cases with VSD, pulmonary stenosis/atresia [61]. Common complications include RV-PA conduit deterioration (Figure 5), necessitating revisions or replacements, coronary artery and pulmonary branch stenosis, and deteriorating subpulmonary RV function due to prolonged pressure. Risks also involve subaortic obstruction and aortic valve dysfunction post-procedure [40,45]. CMR scans are essential for evaluating ventricular function, conduit and aortic baffle conditions, and coronary artery patency, using 2d bSSFP, angiography, and PC flow MRI to detect and quantify stenosis and regurgitation [40].

#### 2.2.2. Congenitally Corrected Transposition of Great Arteries (cc-TGA)

cc-TGA is a rare congenital cardiac malformation known as “double discordance”, characterized by atrio-ventricular and ventriculo-arterial discordance, representing less than 1% of all CHDs [62,63,64,65,66,67]. CMR is the preferred method for assessing the sRV [12,66] and TR for planning valve interventions, as well as for identifying myocardial fibrosis, which impacts sRV function over time [12,45,55]. The role of CMR extends to presurgical planning and monitoring post-surgical outcomes, helping to visualize ventricular function, anatomical repairs, and potential complications [40]. In 2019, Kawakubo et al. introduced the use of fractal analysis with CMR feature tracking to assess RV remodeling and myocardial strain, which could serve as indicators of systemic afterload response in adults with cc-TGA [66].

#### 2.2.3. Tetralogy of Fallot

**Figure 6:**
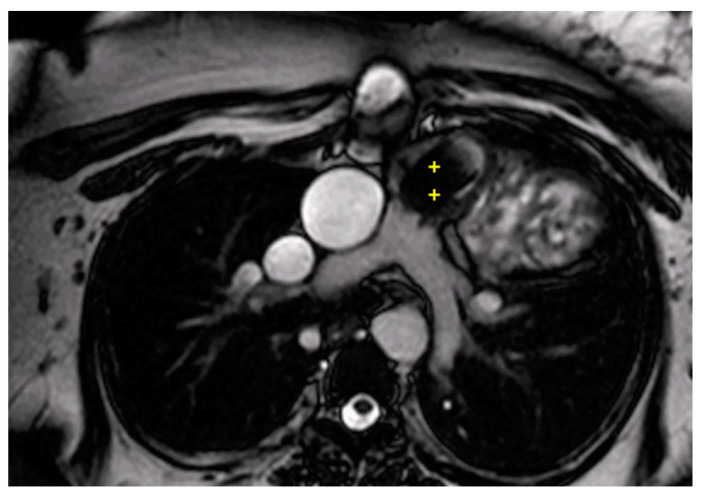
Cine bSSFP image from a 15-year-old male with TOF, post complete correction and Melody valve implantation. The image shows significant migration of the Melody valve into the infundibulum (++).

TOF is a key type of CHDs, comprising 5 to 7% of all CHDs and it requires ongoing comprehensive management across a patient’s life [67]. CMR is essential for the longitudinal monitoring of TOF, offering detailed insights into cardiac morphology, function, and hemodynamics, and is less invasive compared to catheterization [68]. It effectively identifies post-surgical complications like pulmonary stenosis and regurgitation, right ventricular dilatation, and residual ventricular septal defects (Figure 6) [65]. CMR is particularly crucial for accurately measuring pulmonary regurgitation, helping to decide the timing for pulmonary valve replacement and evaluating myocardial viability for surgical planning [69,70]. Recent advancements, like 4D flow imaging, enhance CMR’s utility by enabling dynamic blood flow visualization and quantification, which is vital in assessing repaired TOF patients, as shown in systematic reviews and studies focusing on valve function and myocardial fibrosis [71,72,73].

#### 2.2.4. Double Outlet Right Ventricle

Double outlet right ventricle (DORV) is characterized by both great arteries primarily arising from the right ventricle, representing 1–3% of all CHDs with an incidence of 3–9 per 100,000 live births [74,75,76]. Its classification hinges on the VSD location, arterial positioning, and potential outflow tract obstructions [77,78,79]. Transthoracic echocardiography initially assesses these anatomies, while preoperative CMR is invaluable for detailed visualizations of VSD and the spatial relationships necessary for surgical planning [77,79,80]. Post-surgery, CMR is critical for evaluating late-stage complications in older children and adults, helping assess structural and functional integrity across multiple cardiac components [80,81]. 4D flow imaging has proven effective in estimating right ventricular outflow tract (RVOT) diameters and characterizing cardiac flow dynamics, while computational fluid dynamics provides a deep analysis of cardiovascular dynamics, crucial for optimizing treatment and predicting patient outcomes [79,82,83].

### 2.3. Coarctation of the Aorta

Coarctation of the aorta (CoA) can be difficult to diagnose in utero, even with the advancements in fetal echocardiography, which can sometimes result in excessive false positives. Fetal CMR is emerging as a potent tool to accurately predict severe neonatal CoA issues before birth [84,85,86].

CMR is highly recommended for comprehensive aortic assessment in adolescents and adults, especially for evaluating the extent and severity of aortic narrowing, post-repair complications, and other critical aortic features. Current guidelines suggest regular CMR examinations post-intervention, with intervals of three to five years depending on the underlying condition. For structural and functional analysis, CMR uses 2D bSSFP cine sequences to assess cardiac volumes, mass, and the hypertrophic effects of long-standing hypertension in coarctation cases [87]. CMR angiography helps delineate the cardiovascular anatomy and identify any abnormalities such as constrictions or collateral circulation [88]. The 3D whole heart sequence, which does not require contrast, also contributes to this, and flow analysis can quantify the collaterals [89,90].

Predictive models based on CMR findings suggest that the minimum aortic cross-sectional area, heart rate-corrected deceleration time, and percentage of flow increase are critical predictors of outcomes in CoA patients [91,92].

### 2.4. Cardiovascular Magnetic Resonance Application in the “Univentricular Heart”

The term “univentricular heart” refers to hearts unable to undergo biventricular repair, typically due to having one functional ventricle or two ventricles unable to support separate pulmonary and systemic circulations consecutively. Examples include conditions such as pulmonary or aortic atresia, severe stenosis with a hypoplastic ventricle, hypoplastic left heart syndrome (HLHS), as well as rare conditions like large intramural cardiac tumors and Ebstein anomaly with extensive atrialization of the right ventricular cavity [93].

Surgical intervention for these cases involves univentricular repair through a Total Cavopulmonary Connection (TCPC) operation, which bypasses the ventricular mass in three stages [93,94]. Cardiac magnetic resonance imaging (CMR) plays a crucial role throughout these stages. Following the Norwood procedure, the decision to proceed to a bidirectional Glenn operation has traditionally relied on echocardiography and diagnostic cardiac catheterization. However, a retrospective study by Muthurangu et al. involving 37 HLHS patients demonstrated that CMR can effectively define ventricular and valvular function, as well as vascular anatomy, aiding in the planning of subsequent surgical interventions [95,96].

Furthermore, Brown et al. conducted a prospective, randomized, single-center trial comparing CMR to catheterization in infants’ post-Norwood procedure, showing CMR to be a safe and cost-effective alternative in appropriately selected patients. However, further research is necessary to determine the generalizability of these findings to other centers [97].

Further on, in the lead-up to TCPC completion, CMR aids in patient selection and preoperative assessment of critical information before the final surgery. Currently, there is no consensus on a standardized diagnostic protocol pre-TCPC—some centers rely on cardiac catheterization, despite the associated risks, while others favor CMR or a combination of both. Pujia Banka et al. in their cohort found that catheterization added little clinical value for about half of the patients, with echocardiography often providing incomplete information, suggesting a need for complementary imaging modalities like CMR [98]. Harris’s group highlighted CMR’s non-invasive assessment capabilities, particularly in predicting outcomes based on branch pulmonary area size and flow before the TCPC operation, potentially indicating patients at risk of prolonged hospitalization [99]. In summary, existing literature suggests that cardiac catheterization may be avoidable in select patients with single ventricle physiology before TCPC [100,101].

**Figure 7:**
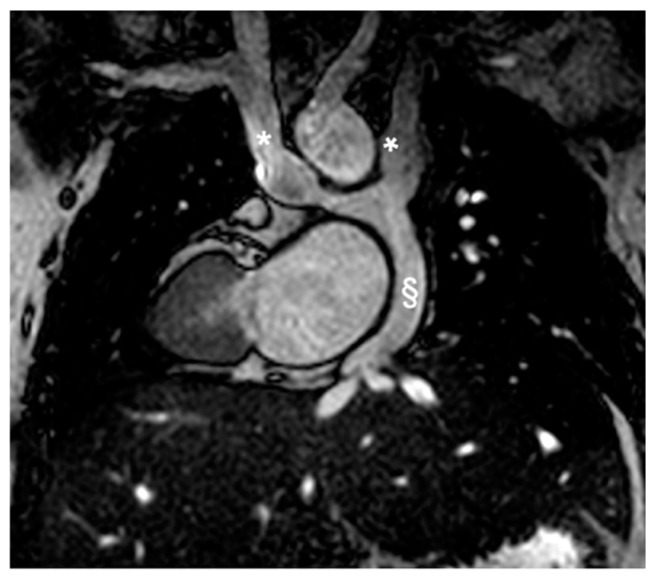
Coronal Cine bSSFP image from a 12-year-old male TCPC with dextrocardia. The image illustrates two superior vena cava (*) and the external conduit (§).

**Figure 8:**
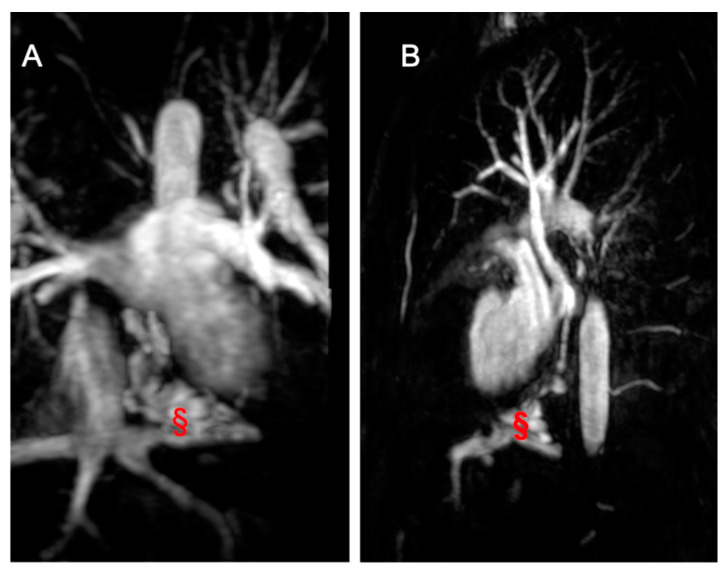
Imaging from a 17-year-old male TCPC. (**A**) shows a coronal angiography image, while (**B**) presents a sagittal angiography image displaying a veno-venous fistula (marked with §) between the suprahepatic veins and the pulmonary veins.

Lastly, CMR plays a crucial role in post-TCPC completion (Figure 7) by providing comprehensive information on anatomy, function, and hemodynamics, essential for identifying and understanding various complications. Routinely used in follow-up, CMR is performed every three to five years, with additional scans conducted when clinically indicated or during emergencies [102,103]. 2D bSSFP cine images facilitate the assessment of wall motion abnormalities, systolic impairment, and volume calculations [104]. Atrioventricular valve regurgitation, a common TCPC complication, is detectable and quantifiable through flow sequences. Moreover, CMR aids in identifying ventricular obstructions and stenosis in pulmonary arteries, systemic veins, and pulmonary veins. Flow sequences present flow distribution patterns of caval flows and pulmonary arteries, providing valuable information supporting potential transcatheter or surgical reinterventions [104,105,106]. Thromboembolic complications are assessed using EGE sequences, particularly important in TCPC patients with atrial arrhythmias. Desaturation can stem from conduit fenestration, pulmonary-to-systemic venous collaterals, or arterial venous malformations (Figure 8). CMR allows the precise calculation of collateral flow contribution to systemic cardiac output, guiding interventions if necessary. Additionally, CMR can investigate TCPC-associated liver disease and lymphatic dysfunction, though specialized protocols may be required. As awareness of the long-term effects grows, further studies will be needed to comprehensively understand TCPC’s impact on other systems [104,105,106].

### 2.5. Evaluation of Coronary Anatomy and Stress Perfusion Imaging

Coronary artery abnormalities (CAA) are uncommon congenital defects, with an estimated prevalence of 1%, involving either anomalous locations of the coronary ostium or abnormalities in the coronary course [107]. These can occur alone or alongside complex CHDs [108,109]. Clinical presentations in children vary significantly, ranging from no symptoms to severe complications like chest pain, ventricular dysfunction, and sudden cardiac death. Post-surgical scenarios, such as after ASO operations for TGA, may necessitate CA evaluations due to complications, likewise coronary allograft vasculopathy (CAV), a notable risk following heart transplantation that negatively impacts long-term outcomes. While invasive coronary angiography remains the gold standard, CMR with vasodilator-infused perfusion has proven effective for detecting anomalies and inducible myocardial ischemia [110,111].

CA aneurysms (CAA), a complication in 15–25% of untreated Kawasaki Disease cases, can progress to rupture, thrombosis, or stenosis, potentially leading to myocardial infarction [112,113]. CT scans provide detailed visualization of CA’s origin and course with excellent spatial resolution, although radiation concerns persist, especially in children [114]. CMR offers a valuable alternative, enabling comprehensive assessments of cardiac structures and functions without radiation. It can identify myocardial edema with T2-weighted images (STIR and T2 mapping) and detect fibrosis with T1-weighted images (LGE), differentiating between ischemic and non-ischemic damage [115].

Despite its advantages, CMR’s longer acquisition times and difficulty distinguishing artifacts from true pathological changes limit its clinical use [116,117]. However, according to European guidelines (Class I, Level C), and a recent American Heart Association statement, CMR is recommended over CT for non-invasive assessment of CAA in young patients, avoiding ionizing radiation [118,119]. Stress sequences using physical or pharmacological agents enhance CMR’s diagnostic capabilities, enabling detailed visualization of myocardial perfusion and ischemia under stress conditions [14,120,121,122]. Although challenges remain in visualizing distal coronary segments and acquiring cooperative patient behavior without sedation, CMR’s comprehensive capabilities make it a preferred modality in pediatric cardiology.

## 3. Challenges and Limitations

There are some considerations that need to be done regarding CMR imaging in the pediatric population. The smaller body size of these patients may require voxel size optimization to maintain an adequate spatial resolution. Technical adjustments to increase signal-to-noise ratio may require longer acquisition time, often not tolerated in pediatric population, particularly under anesthesia. Similarly, the higher heart rates may hamper temporal resolution and require specific adjustments in several sequences at a cost of an increase in scan time. Young children may also require anesthesia or sedation; this is generally safe when performed by experienced staff, but nevertheless it requires additional coordination between different departments and may be unavailable in smaller centers. In addition, risks of adverse events still exist, particularly in patients with cardiomyopathies, severe CHDs, and pulmonary hypertension. Acquiring CMR cine images usually requires appropriate breath hold, which can be addressed by using free-breathing techniques [123]. Concern arises about repeated use of GBCA, often required in follow-up scanning, due to the evidence of gadolinium deposition within the brain [124]. More recently, ferumoxytol, a superparamahnetic iron oxide particle, has emerged as an alternative to GBCA with encouraging safety data also in the pediatric population [125]. Finally, limitations to the use of CMR may stem from its relatively high cost compared to other cardiac imaging techniques, as well as from the limited availability of the technology and of the specialized training required for its application in CHD and in the prenatal diagnosis [126].

## 4. Conclusions and Future Directions

CMR is an advanced cardiovascular imaging tool crucial for diagnosing and managing CHDs. It enables precise assessments of cardiac anatomy, function, hemodynamics, and tissue characteristics, and is particularly effective for complex cases due to its 3D capabilities [11,13,18,19,110]. The leading role of CMR in the challenging management of CHD is confirmed by both the American College of Cardiology/American Heart Association (ACC/AHA) and the European Society of Cardiology (ESC), which emphasize the use of CMR in the initial evaluation of patients with particularly complex anatomical structures and for the serial evaluation of patients at risk of RV enlargement and dysfunction [127,128]

These statements are also supported by two expert consensus documents from radiologists and cardiologists that outline the appropriateness criteria for the use of CMR use in various clinical contexts, including CHD [129,130].

Looking ahead, CMR is poised to integrate further with technologies like artificial intelligence (AI), which enhances the automation of image analysis and the development of predictive models to optimize personalized treatments and outcomes, despite some existing limitations. The application of deep-learning in CMR imaging acquisition appears very promising, as it enables automated localization and detection of the heart, thereby reducing the long acquisition times. Furthermore, AI helps in shortening the time needed for exam evaluation by facilitating image reconstruction with advanced reconstruction function, and improving the post-processing phase, particularly in the CMR segmentation and the automatic characterization of myocardial tissue [131,132,133,134,135,136,137,138,139,140,141,142].

Additionally, the growing application of CMR in prenatal cardiology suggests its future integration into routine prenatal screening for high-risk pregnancies, potentially revolutionizing early CHDs detection and management [117,118,119]. With advancements in 3D modeling and virtual reality, CMR will continue to enhance presurgical planning and educational tools in cardiology, making it an indispensable resource in the evolving landscape of congenital cardiac care [81,82,138,139,140,141,142].

## Figures and Tables

**Table 1:** Advantages and disadvantages of echocardiography and cardiovascular magnetic resonance (CMR). * Unless performed in expert centers where non-MRI conditional devices are performed in adults.

Echocardiography	Cardiovascular Magnetic Resonance
***Advantages***	***Advantages***
Low cost Widely available Radiation free	Radiation free Gold standard for volumetric assessment Provides complimentary tissue characterization
***Disadvantages***	***Disadvantages***
Specialized training/specialized center in CHD echocardiography Limited image quality in poor acoustic windows High operator dependence	Limited availability: specialized/research center for CHD CMR Contraindicated in patients with non-conditional devices * Longer acquisition time and cooperation (breath-holding) required Potential reaction to contrast agents

## Abbreviations

2D	Two-Dimensional
3D	Tri-Dimensional
4DFlow CMR	Four-Dimensional Flow CMR Resonance
AI	Artificial Intelligence
ASDs	Atrial Septal Defects
ASO	Arterial Switch Operation
AtSO	Atrial Switch Operation
AVSDs	Atrioventricular Septal Defects
BSA	Body Surface Area
bSSFP	balances Steady-State Free Precession
BT	Blalock-Taussig
CA	Coronary Arteries
CAA	Coronary Artery Abnormalities
CAV	Coronary Allograft Vasculopathy
cc-TGA	Congenitally Corrected Transposition of Great Arteries
CoA	Coarctation of the aorta
CtA	Conotruncal Anomalies
CFD	Computational Fluid Dynamics
CCHD	cyanotic CHD
CHD	Congenital Heart Diseases
CS	Circumferential Strain
CMR	Cardiovascular Magnetic Resonance
CCT	Cardiovascular Computed Tomography
D-TGA	Dextro-Transposition of Great Arteries
DORV	Double outlet right ventricle
ECG	Electrocardiogram
EF	Ejection Fraction
EGE	Early Gadolinium Enhancement
FT-GLS	Feature Tracking-Global Longitudinal Strain
GBCA	Gadolinium-Based Contrast Agents
HLHS	Hypoplastic Left Heart Syndrome
HFpEF	Heart Failure with preserved Ejection Fraction
IAA	Interrupted Aortic Arch
IVC	Inferior Vena Cava
KD	Kawasaki Disease
LGE	Late Gadolinium Enhancement
LV	Left Ventricle
LV-EDV	left ventricular end diastolic volume
NYHA	New York Heart Association
MRA	Magnetic Resonance Angiogram
MRI	Magnetic Resonance Imaging
PA	Pulmonary Artery
PDA	Patent Ductus Arteriosus
PC	Phase Contrast
Qp/Qs	pulmonary-to-systemic circulation flow ratio
r-TOF	repaired Tetralogy of Fallot
RV	Right Ventricle
RVOT	Right Ventricle Outflow Tract
sRV	Systemic Right Ventricle
SVASD	Sinus Venosus Atrial Septal Defect
SVC	Superior Vena Cava
SV	Stroke Volume
TA	Truncus Arteriosus
TCPC	Total Cavopulmonary Connection
TEE	Transesophageal Echocardiography
TGA	Transposition of Great Arteries
TOF	Tetralogy of Fallot
TTE	Transthoracic Echocardiography
VSDs	Ventricular Septal Defects
